# Can we learn from the ecology of the Bohemian gentian and save another closely related species of Gentianella?

**DOI:** 10.1371/journal.pone.0226487

**Published:** 2019-12-19

**Authors:** Zdenka Křenová, Jiří Brabec, Sabine Rössler, Pavel Kindlmann

**Affiliations:** 1 Global Change Research Institute CAS, Department of Biodiversity Research, Brno, Czech Republic; 2 Charles University, Faculty of Science, Institute for Environmental Studies, Praha 2, Czech Republic; 3 University of South Bohemia, Faculty of Science, České Budějovice, Czech Republic; 4 Museum Cheb, nám. Krále Jiřího z Poděbrad 493/4, Cheb, Czech Republic; 5 Falkenhorstweg 12, München, Germany; Institute for Applied Ecology, UNITED STATES

## Abstract

Bohemian gentian (*Gentianella praecox* subsp. *bohemica*) is an endemic taxon that occurs on the Czech Massif and together with the Sturmian gentian (*Gentianella obtusifolia* subsp. *sturmiana*) are the only autumnal species of *Gentianella* with large flowers in central Europe. Both species have declined dramatically in both population size and numbers of populations. The Bohemian gentian rescue programme, which recommended appropriate management measures, was adopted in 2011. Here we study the ecology of this species, results of the rescue programme and explore the possibilities of using the experience resulting from this programme for improving the viability of the second species. Long-term monitoring of populations of the Bohemian gentian has shown that regular mowing or grazing together with careful litter removal and gap creation are necessary for its survival in the current climatic conditions. We found some ecological differences between these two closely related species of *Gentianella*. However, our empirical experience of the largest population of the Sturmian gentian at a site where it thrives, and general evidence that gaps are crucial for the successful establishment of *Gentianella* seedlings, indicate that regular mowing or grazing together with careful litter removal and creation of gaps, should also be recommended as in the case of the Bohemian gentian rescue programme. Artificial gaps are especially crucial for successful seedling regeneration in oligotrophic meadows with dense vegetation, where the last Sturmian gentian populations survive.

## Introduction

Habitat destruction and fragmentation have restricted an increasing number of plant species to small and isolated populations. Together with various orchid species [1,2,3,4] many species of *Gentiana* [5,6,7] and *Gentianella* [8,9] have also dramatically declined in terms of both the number and size of their populations. Some have already gone extinct and many others only survive in a few isolated populations. They are listed in the IUCN Red List (http://www.iucnredlist.org), are strictly protected in countries of their occurrence, and some of them are in Annex II of the Habitat Directives 92/43/EEC [10]. Genetic studies have shown that most species within the genus *Gentianella* occurring in Europe are closely interrelated [11, 12]. The species are strongly dependent on traditional extensive mowing and grazing, and negatively reflect land use changes that are now commonly occurring. Therefore, they have disappeared from both most intensively managed and abandoned localities. Consequently, conservation projects were implemented in many countries. Successful rescue of rare species can be affected by many factors [13,14] and it is challenging to optimise the management of a species surviving in a few small populations.

Bohemian gentian (*Gentianella praecox* subsp. *bohemica*) is an endemic taxon on the Czech Massif and together with the Sturmian gentian (*Gentianella obtusifolia* subsp. *sturmiana*) are the only two surviving species of autumnal flowering *Gentianella* with large flowers in central Europe. Both species have dramatically declined in terms of both the number and size of their populations. Herbarium reviews have revealed samples of the Bohemian gentian from more than 700 sites, but now this species survives in only 122 sites [15,16]. Similarly, more than 150 locations of the Sturmian gentian in the Bohemian region are recorded in herbaria [16], but now this species is only present at ten sites [17,18]. Both species are strictly protected in all countries within their natural distributions, including the Czech Republic, but only the Bohemian gentian is listed in Annex II species of the Habitat Directives 92/43/EEC.

*Gentianella praecox* subsp. *bohemica* does not have any narrow link to certain types of vegetation [15]. The species grows mainly on pastures, submontane and mountain grassland meadows (*Cynosurion*, *Violion caninae* and *Nardion* phytosociological association). It further grows in some types of mezic hay meadows, in desiccating wetland meadows (*Arrhenatherion* and *Molinion* ass.) and in some broad-leaved dry grasslands and pastures (*Bromion erecti* and *Koelerio-Phleion phleoidis* ass.). The Bohemian gentian also grows in many disturbed habitats (for example at roadsides, quarry and forest edges or at military training sites.

*Gentianella obtusifolia* subsp. *sturmiana* prefers more specific habitats than most of central European gentians [17,18,19]. As far we can infer from recent distribution and historical records, most populations of this species occur in wet, semi-humid or mesic pastures and hay meadows (usually *Molinion*, *Cynosurion* and *Arrhenatherion elatioris* phytosociological ass.) with partial overlap to short grasslands (*Violion caninae* ass.). Presumably, the Sturmian gentians could have also colonized (after some local disturbances, i.e. grazing, gap creation) nutrient-rich communities, both on wet habitats (*Caricion davallianae* ass.) and on dry hillsides (*Bromion erecti* ass.). *Gentianella obtusifolia* subsp. *sturmiana* has probably never grown in nutrient-poor habitats on acidic substrates [17,18,19]. Until now, the populations of the Sturmian gentian have survived only in localities, where more or less regular grassland management with gaps creation was preserved, i.e. in wet pastures in stream floodplains, around meadow springs and on sites periodically affected by grazing.

Our long-term experience with management and monitoring of the Bohemian gentian locations allows us to evaluate the quality of a location. Number of flowering plants, occurrence of seedlings, description of vegetation (species composition, vegetation structure, litter and gaps cover) and gaps occurrence are crucial parameters for evaluation of population vitality and prediction of future status. The Bohemian gentian rescue programme (BGR programme) [15] with recommended appropriate management measures for this species was prepared by the Czech Ministry of Environment and adopted in 2011.

BGR programme has defined the following management measures for Bohemian gentians locations:

- Removal of biomass at wet and mesic sites at the time of the highest increase in growth biomass by mowing or grazing (May to beginning of June, mid-June at the latest);
- Removal of biomass by mowing or grazing when the plants ripen and drop seeds (after 15 October or early spring);
- Creating of gaps for the germination of seeds by disturbing turf (with harrowing, verticulation or raking) or by grazing after ripening and dropping of seeds (after 15 October, or before 20 April),

It is emphasized in the BGR programme that depending on the vegetation type, site conditions, site location, number of flowering specimens and actual weather in the season, it is necessary to modify the primary time schedule and intensity of the measures.

Currently there is no rescue programme for the Sturmian gentian. Because of the similarity of these species, it is likely that the conservation measures suitable for one of them may also be suitable for the other. Therefore, in this paper, we evaluate the conservation implications of the BGR programme and explore the possibilities of using the experience resulting from the BGR programme for increasing the viability of the Sturmian gentian populations. Our results will be used as a basis for preparing the Sturmian gentian rescue programme.

## Methods

### Study species

Two *Gentianella* species were studied: Bohemian gentian, *Gentianella praecox* (A. et J. Kerner) E. Mayer subsp. *bohemica* (Skalický) Holub, and Sturmian gentian, *Gentianella obtusifolia* (F. W. Schmidt) Holub subsp. *sturmiana* (A. et J. Kerner) Holub [synonymum *G*. *aspera* (Hegetschw.) Dostál ex Skalický, Chrtek et Gill].

#### Species description

Bohemian gentian is (2–)10–25(–75) cm tall with 1–50(–360) flowers and Sturmian gentian is (2–)10–25(–51) cm tall with (1–)3–60(–305) flowers [20]. Both species usually have rich blue-violet, sometimes pale lilac blue, pentamerous corollas (2.0–)2.4–3.0(–3.6) cm long. *G*. *obtusifolia* subsp. *sturmiana* has a 2 cm long calyx with narrow wings sloping down to the stem. The wing edges and medium veins of the calyx are densely papillary. Flowers of *G*. *praecox* subsp. *bohemica* do not have wings sloping down to the stem and the calyx is glabrous, or just slightly hairy. Both species are strictly biennial.

#### Life cycle

Both species flower in autumn (mostly September), followed by seed production. Seeds either germinate the next spring (April, May), or they remain in the seed bank. Germinated seeds slowly develop during the first season into rosettes. One-year-old plants form rosettes that produce flowering stems in the autumn of the following year. Numbers of individuals in populations fluctuate greatly from year to year [18,21,22].

#### Pollination

Both species are pollinated by insects, mainly bumblebees.

#### Distribution

**Fig 1:**
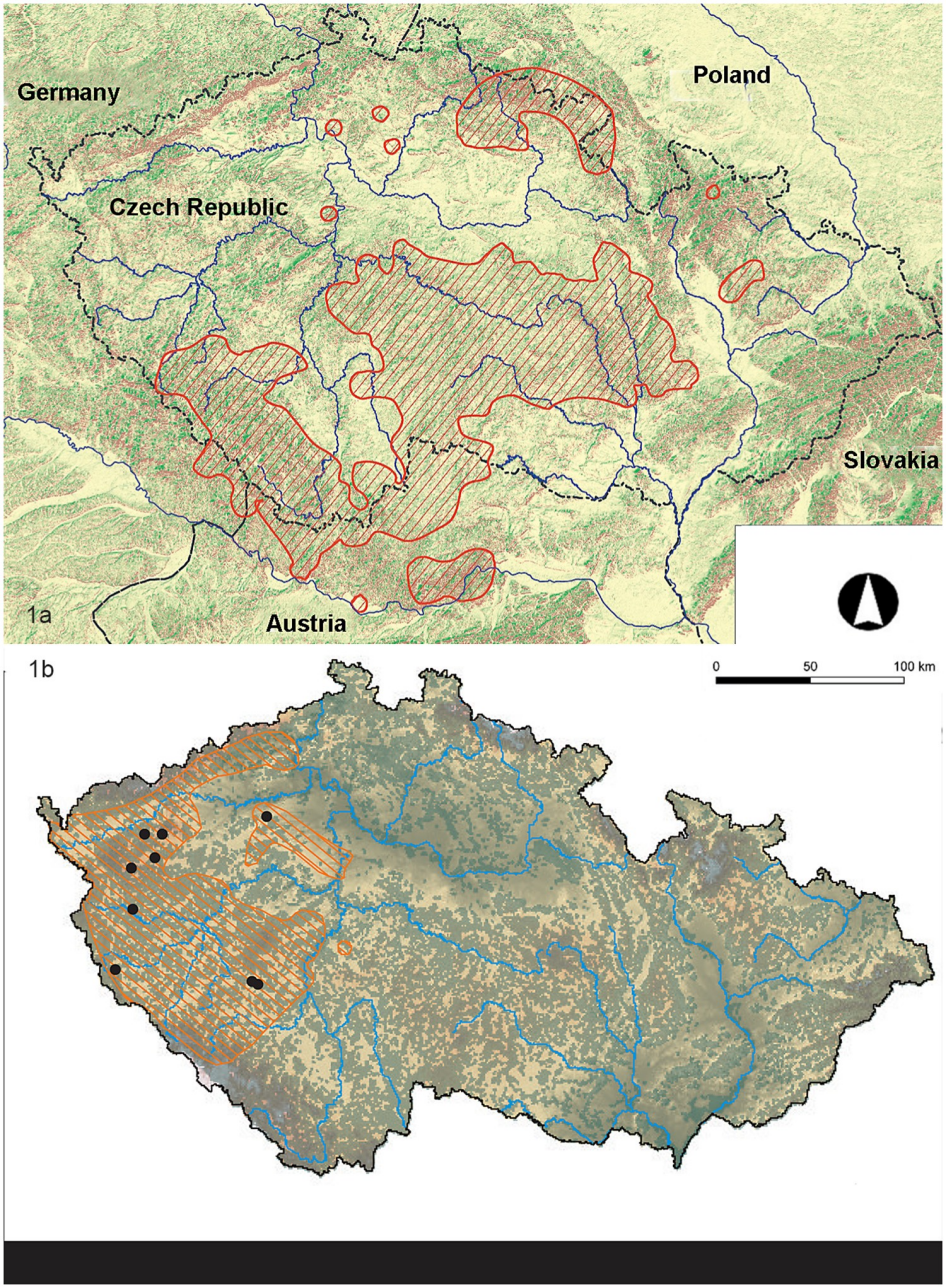
Distribution of studied species. 1a: Historical distribution (red stripes) of *Gentianella praecox* subsp. *bohemica* in central Europe. 1b: Historical distribution (red stripes) and locations where *Gentianella obtusifolia* subsp. *sturmiana* is currently occurring (black dots) in the Czech Republic. Reviews of herbarium specimens and historical records were used to define the historical distribution.

*Gentianella praecox* subsp. *bohemica* is an endemic taxon on the Bohemian Massif. Its historical distribution included the Czech Republic, Bavarian and Austrian part of the Bohemian Forest (Böhmerwald), and the southern part of Poland (Fig 1a) [15]. After 2000, it was found at only 122 locations all over its natural distribution: 74 in the Czech Republic [22], 36 in Austria [23], 8 in Germany [24], and 4 in Poland [25]. *Gentianella obtusifolia* subsp. *sturmiana* occurs in two distinct ranges: one in the northernmost part of the Alps, Hohe Tauren and foothills between the Alps and the Danube River [26]; and the second is located in western and south-western Bohemia, extending to Bavaria, Saxony and Thuringia [18,26]. The Vltava River canyon is the eastern border of the species historical range (Fig 1b). The Sturmian gentian is supposed to be a polyphyletic species [27], which now occurs at several dozen locations. Locations found during this period were Alps [26,28,29] and only a few populations survive in the former species range in the Czech Republic [17,19]. We have records of more than 150 historical sites for *Gentianella obtusifolia* subsp. *sturmiana*, but the occurrence of this species has been confirmed only at ten sites since 2000 there.

#### Life histories and management

Life history strategies and sensitivity of these species to different types of management are recorded only for the Bohemian gentian [21]. Regular mowing or grazing at the right time with careful removal of litter and mosses together with the creation of gaps was found to be the most appropriate management. The regular and/or relatively regular mowing or grazing at the right time with irregular removal of litter and mosses and occasional gap creation also creates a suitable habitat for these species.

### Data collection and analysis

From 1999 to 2019, we repeatedly visited all known localities of the Bohemian and Sturmian gentians and annually recorded numbers of flowering gentians and type of management. If necessary, we asked the site manager for more details. We have recorded the measures applied (e.g. grazing, mowing, digging), time of year, occurrence of litter (yes/not) and gaps (low, patchy, high density) there. Newly discovered locations found later during this period were also recorded. Altogether, we analysed the data for 89 locations of the Bohemian gentian, including 14 in Germany and Austria, and 10 locations of the Sturmian gentian. For calculations, we used averages. We also repeatedly recorded the biotic and abiotic conditions (vegetation relevé, cover etc.) to account for seasonal variability. Vegetation data and soil samples were collected mainly in 2014–2017.

We described the vegetation structure recorded in 5×5 m relevés (Zurich-Montpellier system) and % bryophyte cover (E_0_), % plant cover (E_1_), % shrub cover (E_2_) and % tree cover (E_3_), usually based on one relevé per site. The plots with vegetation relevés were placed in places where gentians occurred. At large or heterogamous sites, two or more relevés were recorded, one at each sub site. Vegetation data were usually recorded at the beginning of flowering of gentians, usually in August. Soil samples were also collected at the same sites and the following parameters were determined by certified laboratories (Institute of Botany of the Czech Academy of Sciences and The Health Institute Pilsen, Laboratory Klatovy): pH, total N, and several ions: Ca^2+^, Mg^2+^, K^+^, exchangeable P. Slope and exposure at all localities were also recorded.

All gentian populations were characterized by their average population size during the study period. We also calculated regression, showing population size in each year, and used the slope of the regression line representing the rate of change in population size of each population in time as a proxy for the trend in population dynamics of the studied populations. For the Bohemian gentian, we calculated these parameters in the years before and after the beginning of the BGR programme.

The owners or site managers managed the sites in different ways both before and after the start of the BGR programme in 2011. We recorded the management applied before and after implementation of the BGR programme and found that some managers fulfilled the BGR programme recommendations and their sites were managed very well and at the right time. Some managers followed recommendations partly and their sites were managed acceptably. Some sites were unmanaged or managed in the wrong way (for example mulching was applied or high stubble with too much litter was found there) and at the wrong time (i.e., in time of growing, flowering of plants or ripening of seeds). To describe the quality of the management, we distinguished five types of management:

Type 1 the most appropriate management, i.e. regular and appropriate mowing or grazing (i.e. short stubble with carefully removed hay, mosses and litter, grazing with only few ungrazed remnants) applied in a proper time (early mowing or grazing before June 15 or later mowing or grazing after ripening of seeds, usually at the end of October). This management means that in time of gentians germination (April/May), the gentian grasslands are completely without felt old grass and the last year litter, mosses cover less than 25% of the area and the litter and moss layer is less than 1 cm thick everywhere. Also gaps are common and regularly created both naturally, by mowing and grazing, and artificially by raking or verticulation.
Type 2 suitable management, i.e. regular mowing or grazing at the right time but insufficient removal of litter and mosses, and irregular creation of gaps. In time of germination (April/May), these gentian grasslands are completely without felt old grass, but the last year litter and mosses cover more than 25% of the area and the litter and moss layer is not more than 2–3 cm thick in some parts of the location.
Type 3 more or less sufficient management, i.e. relatively regular mowing or grazing, mostly at appropriate times; no litter and moss removal, and no creation of gaps. In time of germination (April/May), felt old grass, usually more than 1 cm thick, coveres most of the gentian grasslands and the litter and moss layer is not more than 3 cm thick.
Type 4 insufficient management, i.e. irregular mowing or grazing or regular management at the wrong time; no litter and moss removal, and no creation of gaps;
Type 5 no management or management applied only at the wrong time and in the wrong way (i.e. mulching).

We used the Canonical Corresponded Analysis (CCA) in the CANOCO package [30] to define major gradients among combinations of explanatory variables in the dataset and to describe the relationships between biological assemblages of species and their environment recorded in Bohemian gentian locations. We studied differences in vegetation structure, environmental conditions, management types, gentian population size and slope of the average population sizes over time. We also used the average population size and slope as explanatory variables and performed a „reverse”analysis (i.e., species composition or soil parameters might influence the population size and its slope, not *vice versa*). In all cases, we tested the significance of the results using the Monte Carlo permutation test. We also used forward selection to choose variables explaining most of the variability.

We used the t-test in STATISTICA 12 [31] to compare the population sizes (i.e. numbers of flowering plants) recorded on the Bohemian gentian locations. Also the soil parameters recorded in soil samples collected from the sites where the Bohemian gentian and Sturmian gentian occur were compared using t-tests. We used ANOVA to test the effect of the different types of management on the sizes of the gentian populations and General linear models in STATISTICA 12 [31] to test the effect of environmental variables on the size of the populations of Bohemian gentians. We used ANOVA, Duncan test, for Sturmiana gentian data from the Kocelovice location and tested if there are significant differences in numbers of gentians growing in sites with different densities of gaps.

## Results and discussion

### Differences between the sites where the Bohemian gentian occurs

Recent populations of the Bohemian gentian occur in several types of grassland. The highest number of populations occur in dry broad-leaved grasslands, which can be classified as phytosociological association *Bromion erecti* and ass. *Koelerio-Phleion phleoidis* and in montane and sub montane meadows and pastures (ass. *Cynosurion*, *Violion caninae* and *Nardion*). Some populations occur in mesic mown meadows and pastures (ass. *Arrhenatherion* and *Cynosurion*) and others in various types of disturbed sites (road or quarry edges, military training areas). Historically, this species occurred also in wet and dried out meadows (ass. *Molinion*), but now this species only occurs at three locations with this type of vegetation. Despite the assignment of the sites to several different phytosociological associations (i.e. vegetation types), there are significant differences in the environmental parameters (i.e. exposition, E_0_, E_1_, E_2_, E_3_, soil pH, N, K+, P, Ca^2+^, Mg^2+^) recorded at the different sites (t-tests, p < 0.001). Forward selection in CCA (Table 1) has shown that the concentration of calcium ions in the soil (Ca^2+^), covers of plants (E_1_), shrubs (E_2_), trees (E_3_) and Type 1 management together explained 12.3% of the cumulative variation in vegetation composition. The effects of other environmental parameters were not significant.

**Table 1:** Results of forward selection in CCA. Environmental variables as explanatory variables of species composition at the sites where Bohemian gentian occurs. Significant p-values are marked by *.

**Statistic**	Axis 1	Axis 2	Axis 3	Axis 4
Eigenvalues	0.13	0.07	0.06	0.05
Explained variation (cumulative)	4,62	6.92	9.02	10.80
Pseudo-canonical correlation	0.82	0.83	0.85	0.88
**Forward Selection Results:**				
Name	Explains %	Contribution %	pseudo-F	p
Ca^2+^	3.80	7.60	2.70	0.000*
E_1_	2.30	4.60	1.70	0.000*
E_3_	2.20	4.50	1.60	0.010*
MangType1	2.10	4.30	1.50	0.000*
E_2_	1.90	3.90	1.40	0.030*

**Fig 2:**
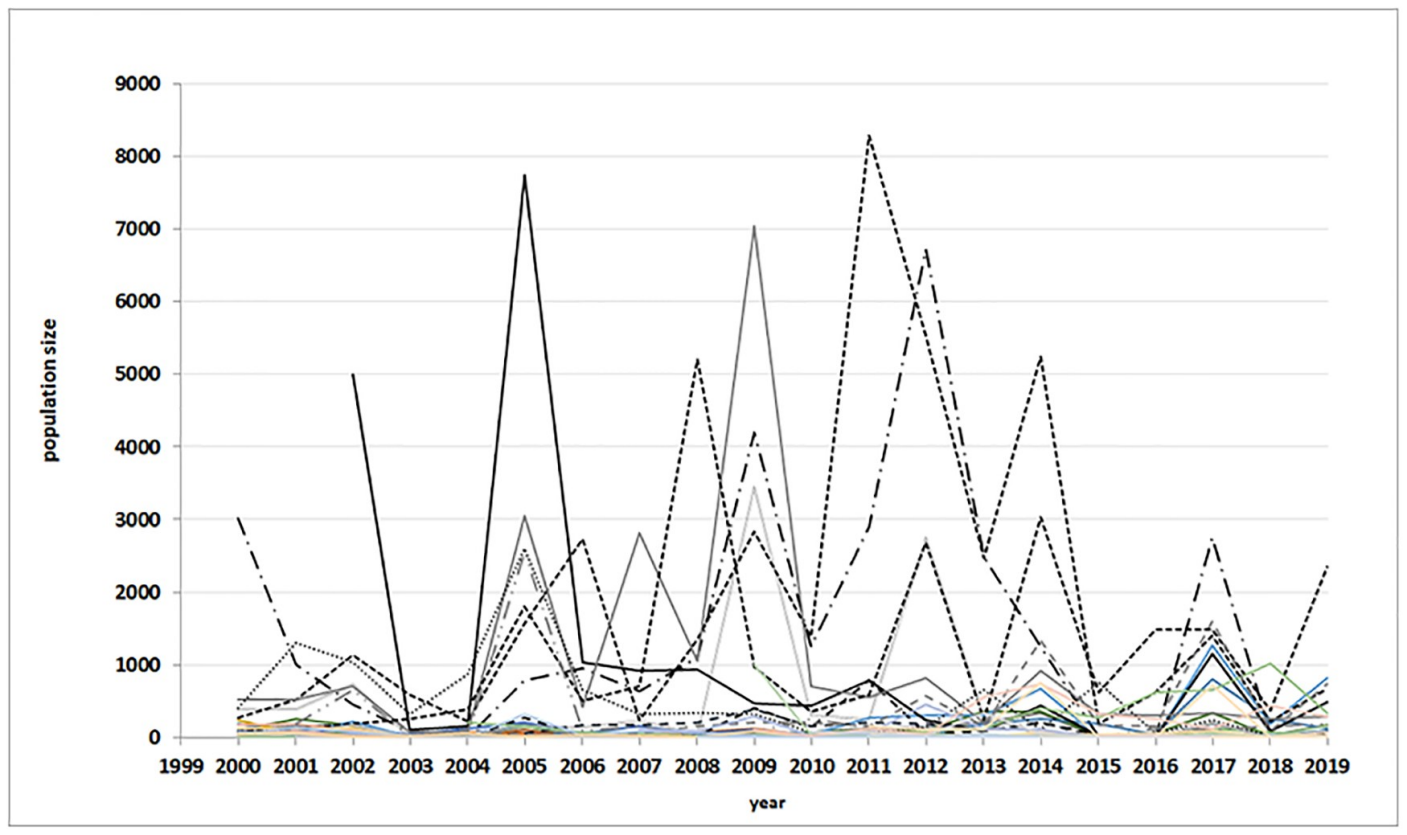
Fluctuation in population sizes of Bohemian gentian over the period 1999–2019. Different lines show different Bohemian gentian populations.

The Bohemian gentian populations differed significantly in the numbers of flowering plants (t-test, d.f. 87, t-value = 4.26, p < 0.001). Many populations consisted of only a few plants, while in several populations there were more than 5,000 individuals in some years. There were big fluctuations in population size over time (Fig 2). Only eight of the populations studied reached an average size of more than 500 plants during the period studied. CCA did not reveal any differences in vegetation structure and other environmental parameters at the different sites where Bohemian gentian occurs and the best predictors of population size were type of management and E_1_ –cover of plants (Table 2).

**Table 2:** Results of GLM analysis. **Effect of environmental variables and type of management on population size of the Bohemian gentian**. Significant p-values are marked *.

Effect	SS	d.f.	MS	F	p
exposition	0.56	1	0.562	0.925	0.341
E_2_	1.38	1	1.378	2.268	0.138
E_1_	3.12	1	3.119	5.133	0.028*
E_0_	0.85	1	0.854	1.405	0.242
pH	0.04	1	0.04	0.065	0.799
N	0.04	1	0.044	0.072	0.789
K^+^	1.74	1	1.739	2.863	0.097
P	0.61	1	0.614	1.010	0.320
Ca^2+^	0.56	1	0.559	0.920	0.342
Mg^2+^	0.84	1	0.837	1.378	0.246
Management type	12.38	4	3.095	5.094	0.002*

### Appropriateness of the type of management included in the Bohemian gentian rescue program

**Fig 3:**
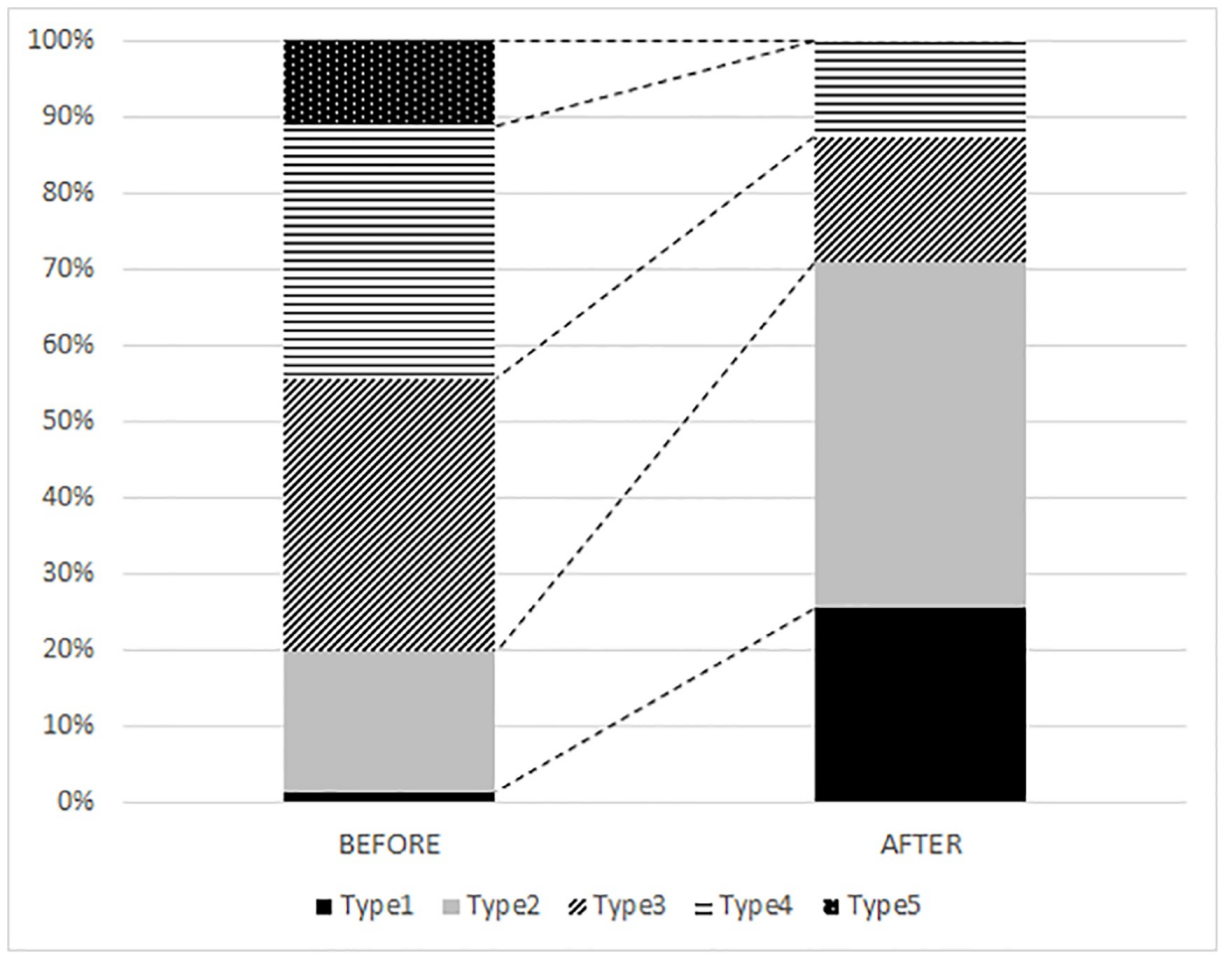
The proportion of sites that were managed with five different management types before and after implementation the Bohemian gentian rescue programme.

Before the start of the BGR programme, Type 1 management was applied at only one of the 87 sites where the Bohemian gentian occurred (Fig 3). Types 2 and 3 management were applied at half of the sites and the remainder were unmanaged (Type 5) or insufficiently managed (Type 4). We used data from only 55 locations to analyse the effect of the BGR programme. Results of the permutation test on all axes: pseudo-F = 1.3. P = 0.002. Twenty-six sites were excluded from this analysis because no flowering gentians had been recorded there during the last eight years of observations, which probably indicates this species is extinct at these sites. These extirpated populations were previously very small, with average population sizes smaller than 15 flowering plants during the years 1999–2009. Also two larger populations, with average population sizes of 40 and 45 flowering plants, went extinct. Both of them were at poorly managed sites, Type 4 and Type 3, respectively.

**Fig 4:**
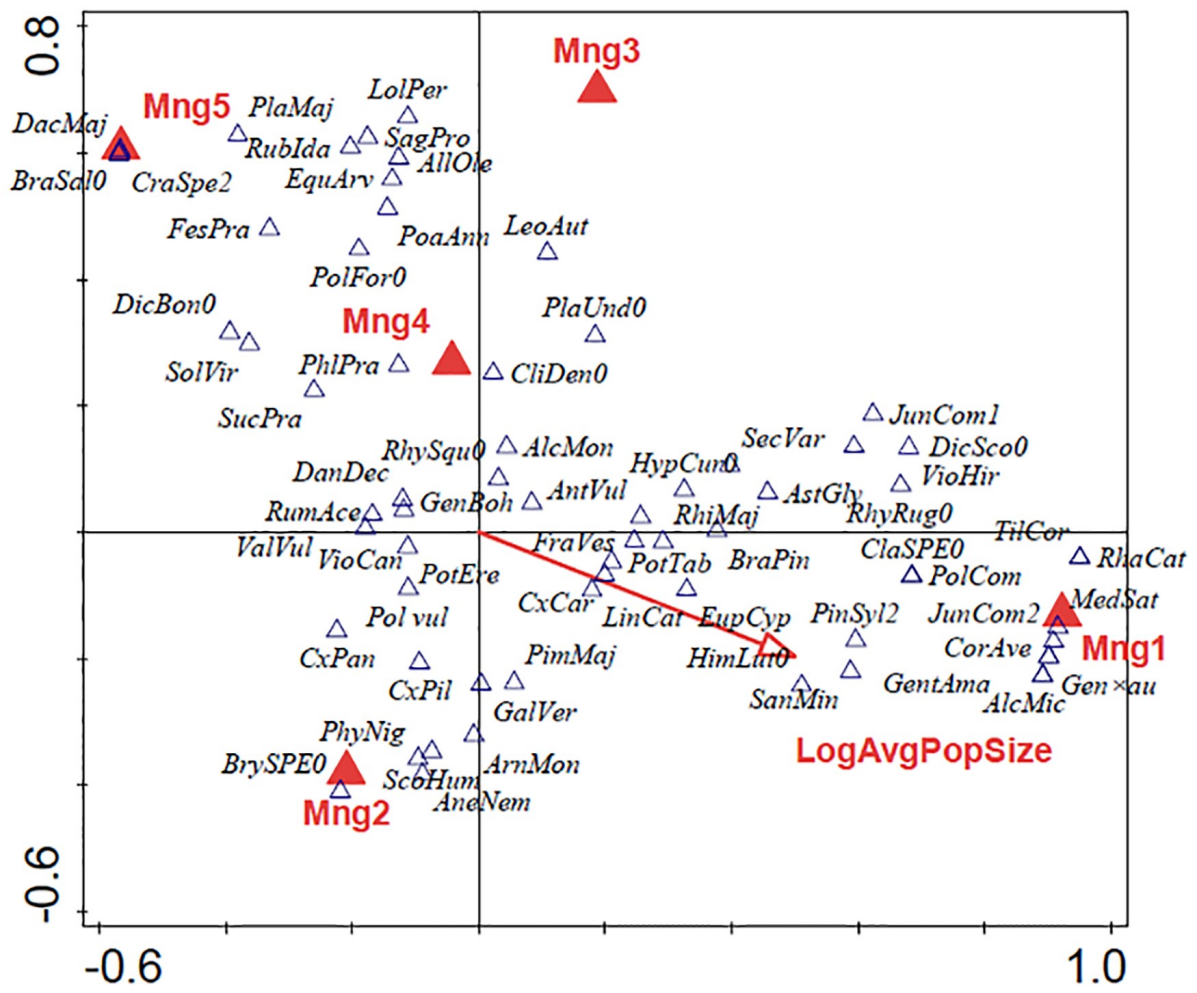
Diagram of the results of the canonical correspondent analysis—Correlation between vegetation composition and type of management and average population size of Bohemian gentian populations. Mng1, Mng2, Mng3, Mng4, Mng5 –types of management; LogAvgPopSize—Average of population size (x+1). Abbreviations of the species names are in Appendix 1.

**Fig 5:**
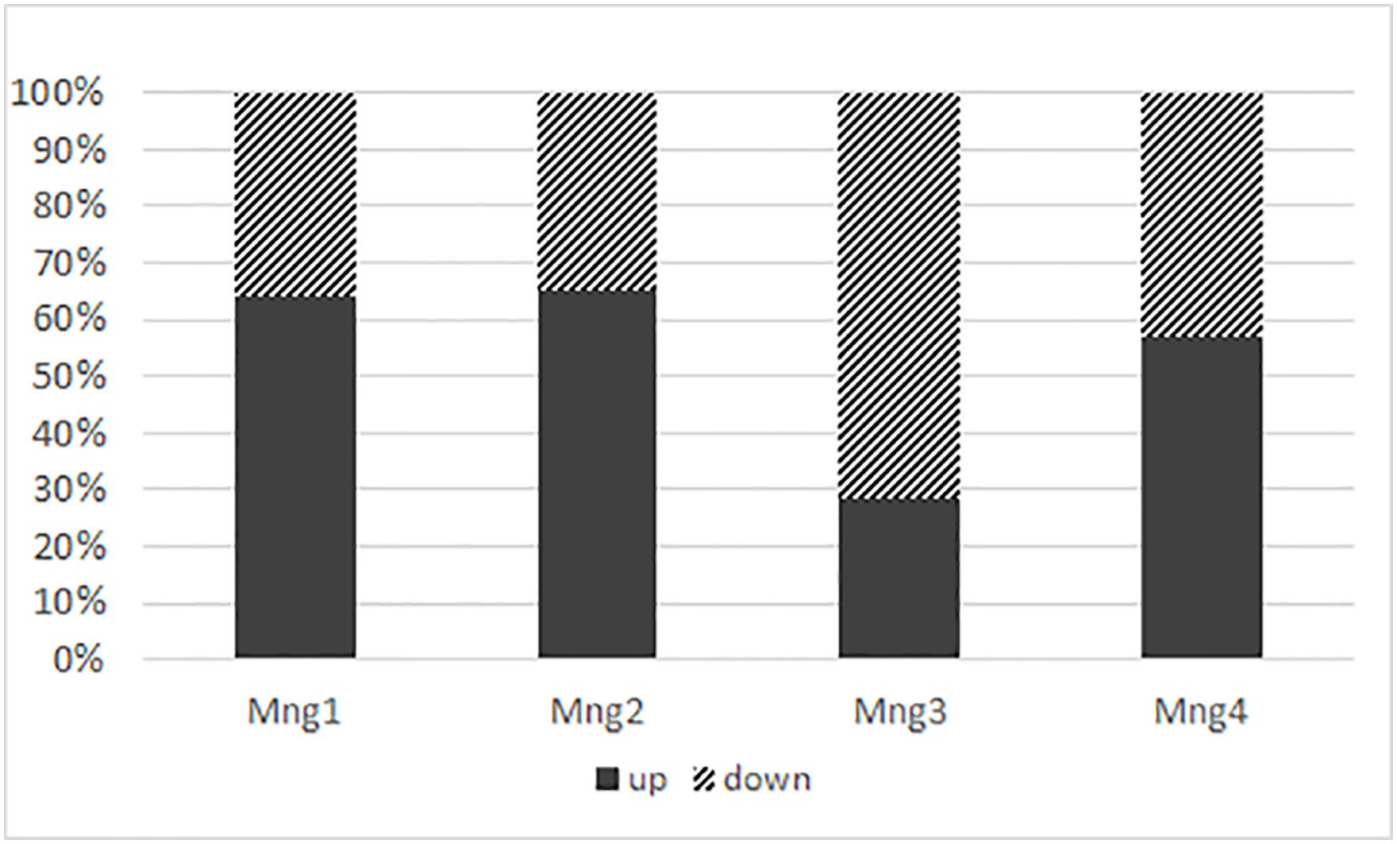
Changes in population slope recorded after the implementation of the BGR programme. Percentage of incidences when the slope, i.e. trend in the size of the population, either increased (“UP”) or decreased (“DOWN”) at the sites with one of four types of management. Mng1, Mng2, Mng3, Mng4 –management Type 1, 2, 3, 4.

Some owners and site managers respected the BGR programme recommendations and some did not. Since the beginning of the BGR programme, no changes in management were recorded at 21 sites and a deterioration in management (from Type 2 to Type 3) at two sites. Management improved at all other sites, with Type 1 management applied at 14 sites and Type 2 at 25 sites, which make up 70% of the 55 locations studied (Fig 3). None of these fifty-five sites were unmanaged (Type 5) and only seven insufficiently managed (Type 4). The significantly largest populations were recorded at sites with Type 1 management and the lowest at sites with Type 5 management (ANOVA, d.f. = 3, F = 8.12, P < 0,001). Similar trends are also revealed by the CCA (Fig 4). Results of the permutation test on all axes: pseudo-F = 1.3, P = 0.002. Higher average population sizes were correlated only with Type 1 management. Despite the fact that the years 2015 and 2016 were not good years for Bohemian gentians, because the extremely dry springs and autumns killed many pre-flowering plants and rosettes, 93% of the populations at sites with Type 1 management had higher average population sizes after implementation of the BGR programme than before. Slopes, used as indicators of a trend in population sizes, improved after implementation of the BGR programme at more than two thirds of the sites with Type 1 management (Fig 5). Negative trends, i.e. calculated slopes < 0, became positive or at least increased at these sites.

The BGR programme has clearly been successful. The best predictors of the population size of this species were the type of management and vegetation cover, the latter being directly affected by the type of management, which improved at many of the sites where Bohemian gentian occurred after the start of the programme. Type 1 management was the most successful followed by Type 2 management. Populations at sites with other types of management declined significantly in size. These new results are less optimistic than previously published results of our field experiment [21]. Results of our sawing experiment indicated that less intensive mowing leaving some litter and mosses and creating only few gaps (analogy of this Type 2 management) could be sufficient for germination of seeds, survival of seedlings and successful survival of the Bohemian gentian populations. This analogy of Type 2 management had only slightly worse results than mowing with careful removing of all hay and litter delivering many gaps (analogy of this Type 1 management). We therefore assume that despite of previously published results, only Type 1 management is likely to support the viability of the populations of Bohemian gentian under current climatic conditions in which climatic extremes are likely to occur more often than in the past.

**Fig 6:**
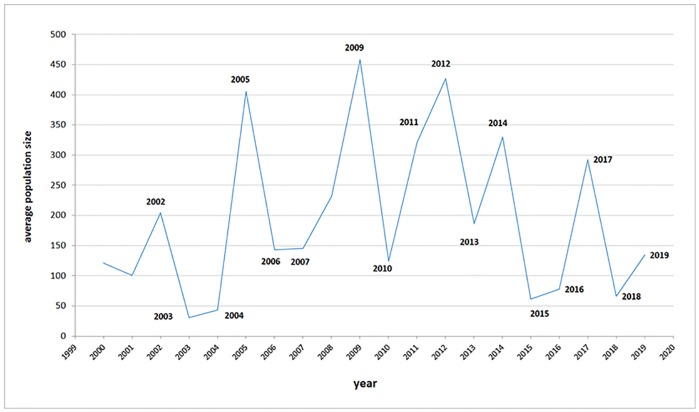
Average population sizes of the Bohemian gentians, calculated as total number of all flowering plants divided by number of locations, where gentians were recorded in that year.

Significant fluctuations in Bohemian gentian population sizes from one year to another can be explained by two intercorrelated factors: (1) Management, discussed in this paper, effected by the site managers and (2) climate conditions, especially lack of precipitations at the time of germination and growing of gentians, which are independent on management but must be reflected in appropriate management measures. A significant rainfall deficit (e.g. during spring and early summer 2003, 2015, 2018) caused dying of many young gentians and reduced numbers of flowering plants in those years. Also many seedlings (leaf rosettes) died and numbers of plants flowering in the next years (i.e. in 2004, 2015, 2019) were reduced. Biennial life history of this species and two-years consequence of each “bad year” can plausibly explain significant declines in years 2003–2004, 2014–2015, 2018–2019 (partly also 2006–2007) at many locations (Fig 6).

Simultaneously, we can assume that the rainfall deficit created gaps in vegetation and these gaps are suitable for germination and growth of gentians from a long-term seed bank. The expectation of increased numbers of flowering gentians was fulfilled in 2005 and only partially in 2017 (Fig 6) for two reasons: (1) weather at the beginning of summer in 2017 was very dry and some smaller plants died in many sites. Drought also caused later flowering of many gentians. (2) there were also some management obstacles in many locations in 2017 and flowing years, which caused lower germination and survival rates. A model study [32] based on experimental population data from five larger populations of the Bohemian gentian collected from 2000–2007 denotes that excessive repetitions of extremely dry seasons can significantly increase a viability of the populations. In the published model [32], frequency of once every three years or more was considered critical in dry years. The question is whether we can approach such frequency of significantly dry vegetation seasons very soon.

### Differences between the sites at which Sturmian and Bohemian gentians occur

Because populations of the Sturmian gentian occur at only a few sites in the Bohemian region, data for only ten populations of Sturmian gentian were analysed. Average population sizes were between less than ten to ten thousand flowering plants per site. Population sizes fluctuate from year to year and fluctuations at the different sites are often synchronised [17]. All these sites are regularly grazed wet pastures with natural gap dynamics, situated on stream alluvia, around meadow springs and near pastures. The composition of the vegetation at sites where Sturmian gentians occur differ slightly from that at sites where the Bohemian gentian occurs. The CCA revealed that the relevance of the vegetation relevé for Bohemian and Sturmian gentians explains only 3.4% of the vegetation composition. The last remaining populations of the Sturmian gentian occur in wet oligotrophic meadows (ass. *Molinion*) and in disturbed and early successional places of a similar type of vegetation. Vegetation at the sites of the two species of *Gentianella* differ slightly. Two groups of *Gentianella* locations were distinguished (Sturmian and Bohemian gentian locations) and inclusion of the locations where the Sturmian or Bohemian gentians occur plus covers of mosses (E_0_), plants (E_1_) and shrubs (E_2_), and are subject to Type 1 management, explained 12.8% of the cumulative variation in the composition of the vegetation (CCA, Table 3).

**Table 3:** Results of forward selection in CCA. Environmental variables as explanatory variables of species composition at the sites where the Bohemian gentian and Sturmian gentian occur. Significant p-values are marked by *.

**Statistic**	Axis 1	Axis 2	Axis 3	Axis 4
Eigenvalues	0.1132	0.0772	0.0505	0.0445
Explained variation (cumulative)	3.43	5.76	7.29	8.64
Pseudo-canonical correlation	0.8749	0.653	0.8026	0.8317
Explained fitted variation (cumulative)	31.91	53.67	67.9	80.44
**Forward Selection Results:**				
Name	Explains %	Contribution %	pseudo-F	p
Sturmian gentian locations	3.4	22.2	3.4	0.002*
Bohemian gentian locations	3.4	22.2	3.4	0.002*
E_1_	1.7	11.2	1.8	0.004*
MngType1	1.7	11.3	1.8	0.004*
E_0_	1.3	8.9	1.4	0.018*
E_2_	1.3	8.9	1.4	0.04*

The soil samples collected at the locations where the Sturmian and Bohemian gentians occur differed significantly in most of the parameters measured. At the locations where the Sturmian gentian occurs the soil had higher pH and levels of calcium ions (Ca^2+^) and magnesium (Mg^2+^) and less potassium (K^+^) and nitrogen (N) than at the locations where the Bohemian gentian occurs (Table 4).

**Table 4:** Results of t-tests. The soil parameters recorded in soil samples collected from the sites where the Bohemian gentian and Sturmian gentian occur were compared. Significant p-values are marked by *.

		Bohemian gentian	Sturmian gentian	F	p
pH	average ± s.d.	5.7±0.8	6.7±0.3	16.89	0.000*
	min–max	4.4–7.7	6.3–7.1		
N %	average ± s.d.	0.5±0.2	0.5±0.1	0.075	0.784
	min–max	0.1–4.1	0.3–0.8		
Mg (mg/1000 g)	average ± s.d.	292.4±226.1	565.7±219.6	14093.00	0.005*
	min–max	2.1–3215	117–845		
Ca (mg/1000 g)	average ± s.d.	2082.8±1475.7	4207.1±3087.5	17868.00	0.001*
	min–max	100.6–8798.3	1400–10400		
K (mg/1000 g)	average ± s.d.	128.5±46.8	74.1±65.9	24016.00	0.002*
	min–max	34–307	0.05–178		
P (mg/1000 g)	average ± s.d.	6.4±3.2	3.9±2.8	5.574	0.020*
	min–max	1.4–19.4	0.5–8.48		

### Can lessons learnt from the study of the Bohemian gentian help protect the Sturmian gentian?

Our analysis has shown that there are some differences between the vegetation and environmental conditions at the sites, where the Sturmian and Bohemian gentians occur. Historically, the majority of the populations of the Sturmian gentian probably occurred in wet oligotrophic, periodically wet or mesic pastures and meadows (ass. *Molinion*, *Cynosurion* and *Arrhenatherion elatioris*) and only a few in short grasslands (ass. *Violion caninae*) [17]. This means that the niche of the Sturmian gentian includes less of Type l managed vegetation than that of the Bohemian gentian [18]. There are also some differences between these two *Gentianella* species in some of their ecological parameters.

Despite of all these differences we believe some lessons from what we know about the optimal management of the Bohemian gentian can be used to benefit the Sturmian gentian for the following reasons. It is well known that seedlings are the most critical part of the life cycle of this species [21,32] and gaps in the vegetation are necessary for the successful germination and survival of seedlings of many *Gentianella* species (e.g. *G*. *campestris* [33], *G*. *germanica* [34], *G*. *praecox* [21]). Whether the gaps have to be artificially created or not depends on the natural frequency of gaps at the typical sites of the species. For example, *Gentianella amarella* subsp. *amarella*, is not so strongly dependent on the creation of artificial gaps, because it grows in dry calcareous meadows with a thin and open vegetation cover that often includes gaps. However, the last of the Sturmian gentian populations typically occur in wet oligotrophic meadows (ass. *Molinion*), which are sensitive to management and when abandoned their biodiversity rapidly declines as the newly dominant species can often produce a lot of biomass [35,36]. A dense vegetation and litter layer can prevent seedling establishment in these meadows and those species entirely dependent on seed reproduction, including gentians, are eliminated. *Gentiana pneumonanthe*, which also occurs in the same type of meadows (ass. *Molinion*), is a good example: artificially created gaps are essential for its successful germination and seedling establishment [6]. Therefore, artificial creation of gaps is also likely to be essential for the successful seed germination and survival of the seedlings of the Sturmian gentian in wet oligotrophic meadows. Any other treatment, like regular mowing or grazing without creating artificial gaps, which is the case for those meadows in which the Sturmian gentian went extinct, are unlikely to be sufficient for its survival. In traditionally managed *Molinion* meadows, gaps were artificially created, for example, accidentally during scything, by the hooves of horses pulling a wagon or wheels of cars in damp soil. Grazing animals, both domestic and wild, in trampling wet meadows would also create gaps. Data on gap density in such meadows in the past are scarce and insufficient to test this hypothesis, nevertheless there is good empirical evidence that wild grazers together with small scale natural disturbances (e.g. drought, local erosion, flooding) could naturally create gaps during pre-agriculture period. Later, regular grazing and/or mowing, careful removal of hay and litter together with a high density of gaps work well even for the Sturmian gentian, as is described below.

**Fig 7:**
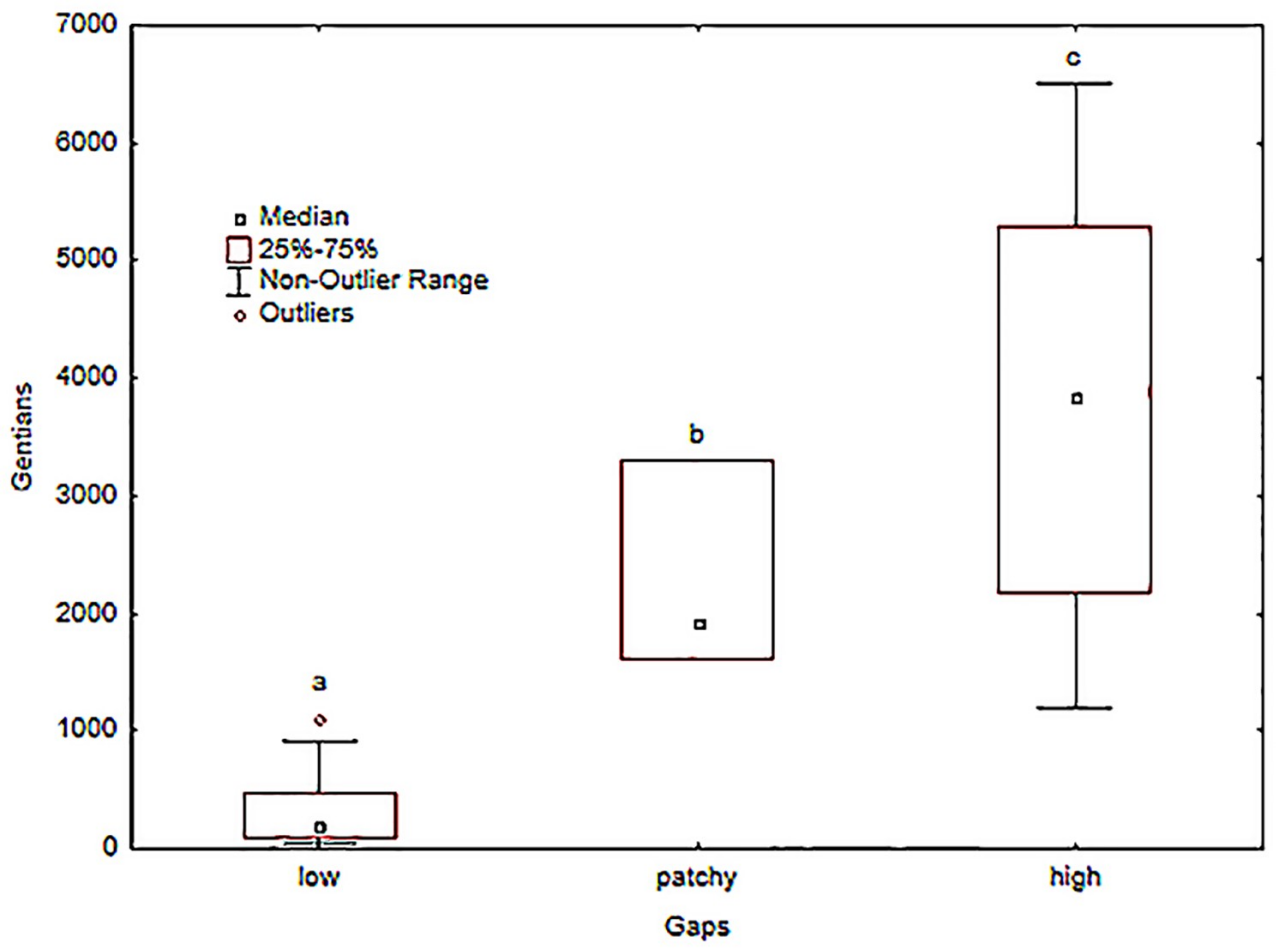
Numbers of plants of the Sturmian gentian (*G*. *obtusifolia* subsp. *sturmiana*), in year t relative to the incidence of gaps in year t − 2. Different letters indicate statistically significant differences in the numbers of plants (Duncan test).

The example is the Kocelovice pasture Nature reserve (Kocelovice pasture NR, 2.29 ha, annually hosting several thousand flowering plants of the Sturmian gentian), which consists of two meadows. The new site manager, who rented this site in 2004, had to remove big stones and level the soil surface so that machines could be used to mow both meadows. This was done in different parts of the NR in different years; therefore the two meadows differed in the density of gaps between years. In some years, it was low and the distribution patchy, and there were only sufficient gaps in a few patches, whereas elsewhere there were many gaps everywhere. We selected data only for this location from our database and found that there were significantly more Sturmian gentians two years after many gaps were created (ANOVA, p<0.001; Fig 7). This is very suggestive, as this species is biennial and gaps are essential for the successful germination and survival of its seedlings.

Results of the long-term monitoring of populations of the Bohemian gentian clearly indicate that regular mowing or grazing, together with careful litter removal and the creation of gaps (Type 1 management) can significantly improve the quality of their habitat. Therefore, this type of management is necessary for the survival of this species under the current climatic conditions. Although the Bohemian and Sturmian gentians differ in some ecological parameters, there are clear indications that Type 1 management is also the best type of management for the Sturmian gentian and should definitely be included in this species rescue programme, which is currently under preparation. For the success of this programme, it is important to stress that the creation of artificial gaps is vital because gaps are essential for successful seedling regeneration.

#### Appendix 1

Abbreviations of the species names used in Fig 4:

**Vascular plants**: AgrCap = *Agrostis capillaris*, AgrSto = *Agrostis stolonifera*, AchMil = *Achillea millefolium* s. l., AjuRep = *Ajuga reptans*, AlcGla = *Alchemilla glaucescens*, AlcMic = *Alchemilla micans*, AlcMon = *Alchemilla monticola*, AlcSpe = *Alchemilla* sp., AllOle = *Allium oleraceum*, AneNem = *Anemone nemorosa*, AntOdo = *Anthoxanthum odoratum*, AntVul = *Anthyllis vulneraria*, ArhEla = *Arrhenatherum elatius*, ArnMon = *Arnica montana*, AstGly = *Astragalus glycyphyllos*, BisMaj = *Bistorta major*, BraPin = *Brachypodium pinnatum*, BriMed = *Briza media*, CamRot = *Campanula rotundifolia*, CarAca = *Carlina acaulis*, CenJac = *Centaurea jacea*, CenSca = *Centaurea scabiosa*, CerHol = *Cerastium holosteoides*, CirAca = *Cirsium acaule*, CirPal = *Cirsium palustre*, CliVul = *Clinopodium vulgare*, CorAve = *Corylus avellana*, CraSpe2 = *Crataegus* sp.–shrub layer, CxBri = *Carex brizoides*, CxCar = *Carex caryophyllea*, CxHir = *Carex hirta*, CxNig = *Carex nigra*, CxPal = *Carex pallescens*, CxPan = *Carex panicea*, CxPil = *Carex pilulifera*, CxTom = *Carex tomentosa*, DacGlo = *Dactylis glomerata*, DacMaj = *Dactylorhiza majalis*, DanDec = *Danthonia decumbens*, DesCea = *Deschampsia cespitosa*, EquArv = *Equisetum arvense*, EupCyp = *Euphorbia cyparissias*, EupRos = *Euphrasia rostkoviana*, FesPra = *Festuca pratensis*, FesRub = *Festuca rubra*, FraVes = *Fragaria vesca*, GalAlb = *Galium album*, GalPum = *Galium pumilum*, GalVer = *Galium verum*, Gen×au = *Gentianella* × *austroamarella* (*G*. *amarella* subsp. *amarella* × *G*. *praecox* subsp. *bohemica*), GenBoh = *Gentianella praecox* subsp. *bohemica*, GenStu = *Gentianella obtusifolia* subsp. *sturmiana*, GentAma = *Gentianella amarella*, HelNum = *Helianthemum nummularium*, HerSph = *Heracleum sphondylium*, HieLac = *Hieracium lactucella*, HiePil = *Hieracium pilosella*, HolLan = *Holcus lanatus*, HypMac = *Hypericum maculatum*, HypPer = *Hypericum perforatum*, JunCom1 = *Juniperus communis*, JunCom2 = *Juniperus communis–*shrub layer, JunCon = *Juncus conglomeratus*, KnaArv = *Knautia arvensis* (incl. *Knautia* × *posoniensis*), KoePyr = *Koeleria pyramidata*, LatPra = *Lathyrus pratensis*, LeoAut = *Leontodon autumnalis*, LeoHis = *Leontodon hispidus*, LeuIrc = *Leucanthemum ircutianum*, LinCat = *Linum catharticum*, LolPer = *Lolium perenne*, LotCor = *Lotus corniculatus*, LuzCam = *Luzula campestris*, LycFlo = *Lychnis flos-cuculi*, MedLup = *Medicago lupulina*, MedSat = *Medicago sativa*, MolCoe = *Molinia caerulea* s. l., NarStr = *Nardus stricta*, ParPal = *Parnassia palustris*, PhlPra = *Phleum pratense*, PhyNig = *Phyteuma nigrum*, PicAbi = *Picea abies*, PimMaj = *Pimpinella major*, PimSax = *Pimpinella saxifraga*, PinSyl = *Pinus sylvestris*, PinSyl2 = *Pinus sylvestris–*shrub layer, PlaLan = *Plantago lanceolata*, PlaMaj = *Plantago major*, PlaMed = *Plantago media*, PoaAnn = *Poa annua*, PolVul = *Polygala vulgaris*, PolCom = *Polygala comosa*, PotEre = *Potentilla erecta*, PotTab = *Potentilla tabernaemontani*, PruVul = *Prunella vulgaris*, RanAcr = *Ranunculus acris*, RhaCat = *Rhamnus cathartica*, RhiMaj = *Rhinanthus major*, RubIda = *Rubus idaeus*, RumAce = *Rumex acetosa*, SagPro = *Sagina procumbens*, SanMin = *Sanguisorba minor*, ScoHum = *Scorzonera humilis*, SecVar = *Securigera varia*, SelCar = *Selinum carvifolia*, SenJac = *Senecio jacobaea*, SolVir = *Solidago virgaurea*, SucPra = *Succisa pratensis*, TarRud = *Taraxacum sect*. *Ruderalia*, ThyPul = *Thymus pulegioides*, TilCor = *Tilia cordata*, TriFla = *Trisetum flavescens*, TriMed = *Trifolium medium*, TriMon = *Trifolium montanum*, TriPra = *Trifolium pratense*, TriRep = *Trifolium repens*, TusFar = *Tussilago farfara*, VacMyr = *Vaccinium myrtillus*, VacVit = *Vaccinium vitis-idaea*, ValVul = *Calluna vulgaris*, VerCha = *Veronica chamaedrys*, VerOff = *Veronica officinalis*, VicCra = *Vicia cracca*, VioCan = *Viola canina*, VioCol = *Viola collina*, VioHir = *Viola hirta*.

**Moses**: AulPal0 = *Aulacomnium palustre*, BraSal0 = *Brachythecium salebrosum*, BrySPE0 = *Bryum* sp., ClaSPE0 = *Cladonia* sp., CliDen0 = *Climacium dendroides*, DicBon0 = *Dicranum bonjeanii*, DicSco0 = *Dicranum scoparium*, HimLut0 = *Homalothecium lutescens*, HylSpl0 = *Hylocomium splendens*, HypCur0 = *Hypnum cupressiforme*, PlaAff0 = *Plagiomnium affine*, PlaCus0 = *Plagiomnium cuspidatum*, PlaUnd0 = *Plagiomnium undulatum*, PleSch0 = *Pleurozium schreberi*, PolFor0 = *Polytrichum formosum*, PsePur0 = *Pseudoscleropodium purum*, RhyRug0 = *Rhytidium rugosum*, RhySqu0 = *Rhytidiadelphus squarrosus*, ThuAss0 = *Thuidium assimile*.
